# Pharmacological Screening Using an *FXN-EGFP* Cellular Genomic Reporter Assay for the Therapy of Friedreich Ataxia

**DOI:** 10.1371/journal.pone.0055940

**Published:** 2013-02-13

**Authors:** Lingli Li, Lucille Voullaire, Chiranjeevi Sandi, Mark A. Pook, Panos A. Ioannou, Martin B. Delatycki, Joseph P. Sarsero

**Affiliations:** 1 Cell and Gene Therapy, Murdoch Childrens Research Institute, Parkville, Victoria, Australia; 2 Bruce Lefroy Centre for Genetic Health Research, Murdoch Childrens Research Institute, Parkville, Victoria, Australia; 3 Department of Paediatrics, The University of Melbourne, Royal Children’s Hospital, Parkville, Victoria, Australia; 4 Department of Clinical Genetics, Austin Health, Heidelberg, Victoria, Australia; 5 Division of Biosciences, School of Health Sciences and Social Care, Brunel University, Uxbridge, United Kingdom; National Institute for Medical Research, United Kingdom

## Abstract

Friedreich ataxia (FRDA) is an autosomal recessive disorder characterized by neurodegeneration and cardiomyopathy. The presence of a GAA trinucleotide repeat expansion in the first intron of the *FXN* gene results in the inhibition of gene expression and an insufficiency of the mitochondrial protein frataxin. There is a correlation between expansion length, the amount of residual frataxin and the severity of disease. As the coding sequence is unaltered, pharmacological up-regulation of *FXN* expression may restore frataxin to therapeutic levels. To facilitate screening of compounds that modulate *FXN* expression in a physiologically relevant manner, we established a cellular genomic reporter assay consisting of a stable human cell line containing an *FXN*-*EGFP* fusion construct, in which the *EGFP* gene is fused in-frame with the entire normal human *FXN* gene present on a BAC clone. The cell line was used to establish a fluorometric cellular assay for use in high throughput screening (HTS) procedures. A small chemical library containing FDA-approved compounds and natural extracts was screened and analyzed. Compound hits identified by HTS were further evaluated by flow cytometry in the cellular genomic reporter assay. The effects on *FXN* mRNA and frataxin protein levels were measured in lymphoblast and fibroblast cell lines derived from individuals with FRDA and in a humanized GAA repeat expansion mouse model of FRDA. Compounds that were established to increase *FXN* gene expression and frataxin levels included several anti-cancer agents, the iron-chelator deferiprone and the phytoalexin resveratrol.

## Introduction

Friedreich ataxia (FRDA) is an autosomal recessive disorder characterized by neurodegeneration and cardiomyopathy. It is the most common form of hereditary ataxia with an incidence of approximately 1 in 50,000 in Caucasian populations [1]. About 98% of individuals with FRDA are homozygous for an expansion of a GAA trinucleotide repeat sequence within the first intron of the *FXN* gene. The remaining individuals are compound heterozygotes for a GAA expansion and a point mutation, deletion and/or insertion. Pathogenic GAA expansion alleles are in the size range of 60 to more than 1300 repeats. The presence of a GAA repeat expansion results in the inhibition of *FXN* gene expression, reduced levels of full length *FXN* transcript and an insufficiency of the mitochondrial protein frataxin [2]–[5].

Frataxin deficiency results in mitochondrial dysfunction including the loss of iron-sulfur cluster (ISC)-containing enzymes, increased oxidative damage and mitochondrial iron accumulation [6]–[8]. Frataxin has been implicated to function as a multimeric iron storage protein that also possesses ferroxidase activity [9], as an iron chaperone which modulates mitochondrial aconitase activity [10]–[12], as a mediator of iron delivery to ferrochelatase [13] and in the early stages of ISC biogenesis by direct interaction with ISU-type proteins [14]–[17]. The main sites of pathology include the large sensory neurons of the dorsal root ganglia and the dentate nucleus of the cerebellum [18], [19].

The mechanism by which the GAA expansion results in reduced *FXN* gene expression is not clear. There is evidence that suggests that the GAA repeat expansion may form an unusual and stable triple helical non-B DNA structure or DNA/RNA hybrid that impedes transcription elongation [4], [5], [20]. It is now apparent that the GAA repeat expansion generates a heterochromatin-mediated gene silencing effect [21], [22]. Changes in both DNA methylation and histone modification have been described [22]–[28].

Overall, an inverse correlation has been found between the size of the smaller GAA repeat expansion and transcript levels, the amount of residual frataxin produced and the age of onset of disease symptoms [29]–[33]. Heterozygous carriers of a GAA repeat expansion produce about half the normal level of frataxin and are asymptomatic. As the GAA repeat expansion mutation does not alter the coding sequence of the gene, it is hypothesized that any increase in frataxin levels should prove beneficial, while a several-fold increase could be sufficient to halt disease progression.

We were the first group to propose the pharmacological up-regulation of *FXN* gene expression as a novel therapeutic approach for the treatment of FRDA [34]. Unlike pharmacological treatments focused on secondary disease effects by using antioxidant and iron chelation therapy [35]–[37] our approach is aimed at directly addressing the primary issue of frataxin deficiency.

We previously reported the identification of a 188 kb BAC clone (RP11-265B8) containing exons 1–5b of the human *FXN* locus and extensive flanking regions both upstream and downstream of the gene [34]. The clone contains a (GAA)_6_ sequence in the region that undergoes expansion within the first intron of the *FXN* gene. We demonstrated that the genomic insert is able to successfully complement the embryonic lethal phenotype of homozygous *Fxn* knockout mice, indicating that key regulatory elements required for normal expression of the *FXN* gene are present within this clone [38]. The BAC clone was used for the generation of an *FXN-EGFP* genomic reporter construct in which the EGFP gene was fused in-frame immediately following the final codon of exon 5a of the human *FXN* gene [34]. The *FXN-EGFP* genomic reporter preserves the normal location and spacing of many regulatory elements that may be positioned over large distances in the surrounding chromosomal region, and facilitates the recapitulation of normal gene expression patterns [39]. The construct was shown to drive the expression of EGFP from the regulatory elements of the *FXN* locus in cell lines and transgenic mice, with the frataxin-EGFP fusion protein targeted to the mitochondria [34], [40]. A stable BHK-21 cell line containing the genomic reporter fusion was established and used to demonstrate the induction of *FXN* gene expression by hemin and butyric acid [34].

In this study we report the development of a stable human cell line containing the *FXN-EGFP* genomic reporter fusion. The sensitive cellular assay for *FXN* gene expression was used to evaluate pharmacological agents for their ability to modulate *FXN* gene expression and adapted for the high throughput screening (HTS) of a small compound library. The evaluation of hit compounds in cells from individuals with FRDA and in a humanized GAA repeat expansion mouse model of FRDA identified a number of agents that were able to increase *FXN* gene expression and frataxin protein, including several anti-cancer compounds, the iron-chelator deferiprone and the phytoalexin resveratrol.

## Materials and Methods

### Ethics Statement

Lymphoblasts and fibroblasts from individuals with FRDA were obtained from Coriell Cell Repositories (Camden, New Jersey, USA) or collected following written informed consent with approval from the Royal Children’s Hospital Human Research Ethics Committee (project 22009). All procedures involving the treatment of mice were carried out in accordance with the UK Home Office ‘Animals (Scientific Procedures) Act 1986’ under project license PPL70/6716. All efforts were made to minimize suffering.

### Tissue Culture and Transfection

HeLa and HeLa (*FXN-EGFP*) cell lines were maintained in adherent culture in DMEM supplemented with 10% fetal calf serum. Lymphoblasts were maintained in suspension culture in RPMI 1640 supplemented with 20% fetal calf serum. Fibroblasts were maintained in adherent culture in BME supplemented with 10% fetal calf serum. Media contained antibiotics (100 U/ml penicillin and 0.1 mg/ml streptomycin) for routine cell maintenance but were omitted during pharmacological compound assays. Cells were cultured at 37°C in 5% CO_2_ atmosphere.

For transfection studies, HeLa cells were plated in 6-well culture plates at a density of 2×10^5^ cells per well and allowed to reach 50–80% confluency. Cells were transfected with 10 µg of purified BAC DNA (linearized by digestion with *Asc*I and *Bsi*WI) using LipofectAMINE (Life Technologies, Grand Island, NY, USA), at a 4∶1 lipid to DNA ratio, according to the manufacturer’s protocol. For the selection of stable clones, cells were transferred to 96-well flat-bottom plates at two days post-transfection and grown in medium containing 300 µg/ml G418 (Life Technologies). G418-resistant clones were isolated via limiting cell dilution at 100–500 cells per ml. Cells were examined for EGFP expression by fluorescence microscopy.

### Fluorescence *In Situ* Hybridization (FISH) Analysis

Cell cultures were harvested after exposure to colcemid for 4 hours. Chromosome preparations were obtained using standard techniques. Cells were spread onto slides and subsequently denatured by immersion in 70% formamide in 2×SSC at 73°C for 3 minutes. The probe was prepared using purified DNA from RP11-265B8, which was labeled by nick translation with digoxigenin, according to the manufacturer’s instructions (Roche, Mannheim, Germany). The labeled DNA was ethanol precipitated together with human COT1 DNA and resuspended in 50% formamide, 10% dextran sulfate, and 2×SSC to a concentration of 40 ng/µl. The probe was denatured by heating at 75°C for 8 minutes, followed by preannealing at 37°C for 20 minutes. Hybridization was at 37°C for 16 hours followed by washing in 1×SSC at 70°C for 5 minutes. The probe was detected with mouse anti-digoxigenin antibody (Roche) followed by rhodamine conjugated anti-mouse antibody (Rockland, Gilbertsville, PA, USA). The slides were mounted in Vectashield (Vector Laboratories, Burlingame, CA, USA) containing DAPI counterstain.

### Pharmacological Compound Analysis *In Vitro*

HeLa (*FXN-EGFP*) cells were plated at a density of 1×10^4^ cells per ml, lymphoblasts were plated at a density of 1×10^5^ cells per ml and fibroblasts were plated at a density of 5×10^4^ cells per ml in appropriate media in 6-well culture plates. Cultures were incubated overnight prior to removal of growth medium and replacement with fresh medium containing various concentrations of test compounds (**Table 1**). Cultures were incubated for a further 72 hrs. Adherent cells were collected by trypsinization. All cells were washed twice in phosphate-buffered saline (PBS). Assays were performed on triplicate cultures in at least three independent experiments.

**Table 1:** Properties of selected compounds.

Compound	CAS number	Molecular formula	Molecular weight	Supplier^a^
δ-Aminolevulinic acid	106-60-5	C_5_H_9_NO_3_	131.13	Sigma Aldrich
Ascorbic acid	50-81-7	C_6_H_8_O_6_	176.12	Sigma Aldrich
Benzalkonium chloride	8001-54-5	C_22_H_40_ClN	354.02	Sigma Aldrich
Betamethasone	378-44-9	C_22_H_29_FO_5_	392.46	Sigma Aldrich
Bupropion hydrochloride	34841-39-9	C_13_H_18_ClNO	239.74	Sigma Aldrich
Camptothecin	7689-03-4	C_20_H_16_N_2_O_4_	348.35	Sigma Aldrich
Cefdinir	91832-40-5	C_14_H_13_N_5_O_5_S_2_	395.42	Sigma Aldrich
Chlorpropham	101-21-3	C_10_H_12_ClNO_2_	213.67	Sigma Aldrich
Chrysanthemic acid	4638-92-0	C_10_H_16_O_2_	168.23	Sigma Aldrich
Cisplatin	15663-27-1	H_6_C_l2_N_2_Pt	301.1	Mayne Pharma
Clozapine	5786-21-0	C_18_H_19_ClN_4_	326.82	Sigma Aldrich
Convallatoxin	508-75-8	C_29_H_42_O_10_	550.65	Sigma Aldrich
Deferiprone	30652-11-0	C_7_H_9_NO_2_	139.15	Lipomed
Desferrioxamine	70-51-9	C_25_H_48_N_6_O_8_	560.68	Novartis
Dimethyl sulfoxide	67-68-5	C_2_H_6_OS	78.13	Sigma Aldrich
Ethanol	64-17-5	C_2_H_6_O	46.07	Merck
Ferric ammonium sulfate	10138-04-2	FeNH_4_(SO_4_)_2_	266.07	Sigma Aldrich
Ferrous ammonium sulfate	10045-89-3	(NH_4_)2Fe(SO_4_)_2_	284.05	Sigma Aldrich
Hemin	16009-13-5	C_34_H_32_ClFeN_4_O_4_	651.94	Sigma Aldrich
Molsidomine	25717-80-0	C_9_H_14_N_4_O_4_	242.24	Sigma Aldrich
Nimustine	42471-28-3	C_9_H_13_ClN_6_O_2_	272.70	Sigma Aldrich
Phenothrin	26002-80-2	C_23_H_26_O_3_	350.46	Sigma Aldrich
Resveratrol	501-36-0	C_14_H_12_O_3_	228.24	Sigma Aldrich
Rifampin	13292-46-1	C_43_H_58_N_4_O_12_	822.96	Sigma Aldrich
Sulfameter	651-06-9	C_11_H_12_N_4_O_3_S	280.30	Sigma Aldrich

a Lipomed (Arlesheim, Switzerland), Mayne Pharma (Indianapolis, IN, USA), Merck (Darmstadt, Germany), Novartis (East Hanover, NJ, USA), Sigma Aldrich (St. Louis, MO, USA).

### Flow Cytometry

The proportion of EGFP-positive HeLa (*FXN-EGFP*) cells and the relative levels of EGFP expression were measured by flow cytometry. Cells were resuspended in PBS with 2% fetal calf serum. Flow cytometry was performed using a LSR II flow cytometer (Becton-Dickinson, Franklin Lakes, NJ, USA) and analyzed using the FACSDiva Software Package.

### Compound Library

The library screened was the Spectrum Collection (MicroSource Discovery Inc., Gaylordsville, CT, USA). The library contains 2,000 biologically active and structurally diverse compounds that are primarily Food and Drug Administration (FDA)-approved compounds, bioactive compounds or natural products. The compounds are supplied as 10 mM solutions in dimethyl sulfoxide (DMSO). More details of the library can be found at http://www.msdiscovery.com/spectrum.html.

### High Throughput Screening Procedures

High throughput screening procedures were conducted at the Walter and Eliza Hall Institute High-Throughput Chemical Screening Facility (Bundoora, Victoria, Australia).

On Day 1 HeLa (*FXN-EGFP*) cells were seeded into 96-well optical-grade NUNC Cytowell Special Optics microtiter plates (Thermo Fisher Scientific, Rochester, NY, USA) at a density of 10^4^ cells per well in a total volume of 200 µl of culture media (DMEM supplemented with 10% fetal calf serum) using a Multidrop 384 high-speed reagent dispenser (Thermo Electron Corporation, Waltham, MA, USA). Cells were allowed to adhere overnight at 37°C in 5% CO_2_ atmosphere.

On Day 2 each test compound was dispensed robotically from the library into individual wells at a volume of 0.2 µl to achieve a final concentration of 10 µM using a Zymark Mini Staccato system (SOTAX Corporation, Westborough, MA, USA). Cultures were incubated for a further 72 hours. Control rows and columns were set up containing: media only, cells without any test compounds, and cells exposed to the positive control drug cisplatin (3.3 µM) which is known to increase *FXN* gene expression. In addition, parental HeLa cells not containing the *FXN-EGFP* genomic reporter were set up as a measure of background cell fluorescence.

On Day 5 the cells were washed twice with 200 µl of PBS via a MiniTrak IX automated liquid handling system (PerkinElmer, Waltham, MA, USA) and overlaid with 200 µl of PBS prior to the fluorometric measurement of EGFP levels in an Envision 2100 Multilabel plate reader (PerkinElmer) configured for EGFP bottom-plate measurements at multiple locations per well.

### Cell Viability Assay

To correct for variations in cell number from well to well due to possible toxicity of different test compounds a measurement of cell viability was performed. This was done following measurement of EGFP levels using the CellTiter-Blue cell viability assay (Promega, Alexandria, NSW, Australia). Culture medium containing the CellTiter-Blue reagent (resazurin) was added to each well and plates incubated at 37°C for 90 minutes. The signal produced by conversion of resazurin to resorufin is directly proportional to viable cell number and was detected fluorometrically in an Envision 2100 plate reader set up for top-plate measurements. A standard curve of cell number versus viability was established for each run.

### Post High Throughput Screening Data Analysis

All fluorescence readings were exported into a set of Microsoft Excel spread sheets programmed to perform the necessary data analysis. The viability readings were converted to cell numbers for each well using the standard curve described above. The average background fluorescence of parental HeLa cells was subtracted from the EGFP readings of HeLa (*FXN-EGFP*) cell exposed to the chemical library. A correction factor for the number of viable cells was applied to the EGFP levels in each corresponding well. This was compared to the average value obtained from cells not exposed to any test compound to determine the level of change in *FXN* gene expression (represented as fold change in expression). This is represented as:
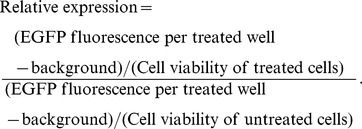



### Pharmacological Compound Analysis *In Vivo*

The YG8R FRDA mice [41] were housed in conventional open cages with Litaspen Premium 8/20 bedding, paper wool nesting and standard fun tunnel environmental enrichment, with 13 hours light, 11 hours dark, 20–23°C and 45–60% humidity. The mice were given a diet of SDS RM3 Expanded food pellets and standard drinking water. YG8R mice at an age of 9–12 months were given subcutaneous injections for three consecutive days of 25–300 mg/kg resveratrol in a vehicle solution of 6.25% DMSO, 20% glycerol, 20% PEG400, 20% propylene glycol, 5 mM sodium acetate (pH 5.2). Each dose of resveratrol or vehicle was evaluated in four mice. After either four hours or 24 hours mice were culled and tissues were collected and snap frozen in liquid nitrogen.

### Quantitative Real-time Reverse Transcription PCR

Total cellular mRNA was isolated from cultured cell lines using the Dynabeads mRNA DIRECT Kit (Invitrogen, Carlsbad, CA, USA) according to the manufacturer’s instructions. Reverse transcription was performed using the SuperScript II First Strand Synthesis System (Invitrogen) with oligo (dT)_12–15_ primers. Real-time PCR was performed on an ABI7300 Sequence Detection System (Applied Biosystems, Carlsbad, CA, USA) using TaqMan Gene Expression Assays (Applied Biosystems) with primers and probes specific for the human *FXN* gene (Hs00175940_m1) and human hypoxanthine phosphoribosyltransferase 1 (*HPRT1*) (Hs99999909_m1) as the reference gene. *FXN* gene expression was determined using the comparative C_t_ method (*ΔΔ*C_t_) relative to the endogenous control *HPRT1*. Assays were performed in triplicate in at least three independent experiments.

Total RNA was isolated from snap frozen YG8R mouse brain tissues by homogenization with Trizol (Invitrogen) and cDNA was prepared by using AMV reverse transcriptase (Invitrogen) with oligo-dT primers. Levels of human transgenic *FXN* expression were assessed by quantitative RT-PCR using an ABI7900 sequencer and SYBR Green (Applied Biosystems) with the primers FRT-I (5′-TTGAAGACCTTGCAGACAAG-3′) and RRT-II (5′-AGCCAGATTTGCTTGTTTGG-3′). Mouse *Gapdh* RT-PCR primers used for normalization were GapdhmF (5′-ACCCAGAAGACTGTGGATGG-3′) and GapdhmR (5′-GGATGCAGGGATGATGTTCT-3′).

### Frataxin Protein Measurements

The levels of frataxin protein were measured by lateral flow immunoassay with the Frataxin Protein Quantity Dipstick Assay Kit (MitoSciences, Eugene, Oregon, USA) according to the manufacturer’s instructions [42]. Signal intensity was measured with a Hamamatsu ICA-1000 Immunochromatographic Reader (MitoSciences).

### Statistical Analyses

For the evaluation of pharmacological compounds in cell lines, the experimental data is reported as the mean ± the standard error of the mean (SEM) of triplicate assays in at least three independent experiments. Comparisons were made between groups of equal size by Student’s t-test for paired data. For the evaluation of pharmacological compounds in mice, reactions were carried out in triplicate for each biological sample (n = 4) and data from the vehicle and drug-treated groups were compared using the Student’s t-test. Data were considered significantly different at *p*<0.05.

## Results

### Generation and Characterization of HeLa (*FXN-EGFP*) Stable Cell Lines

**Figure 1:**
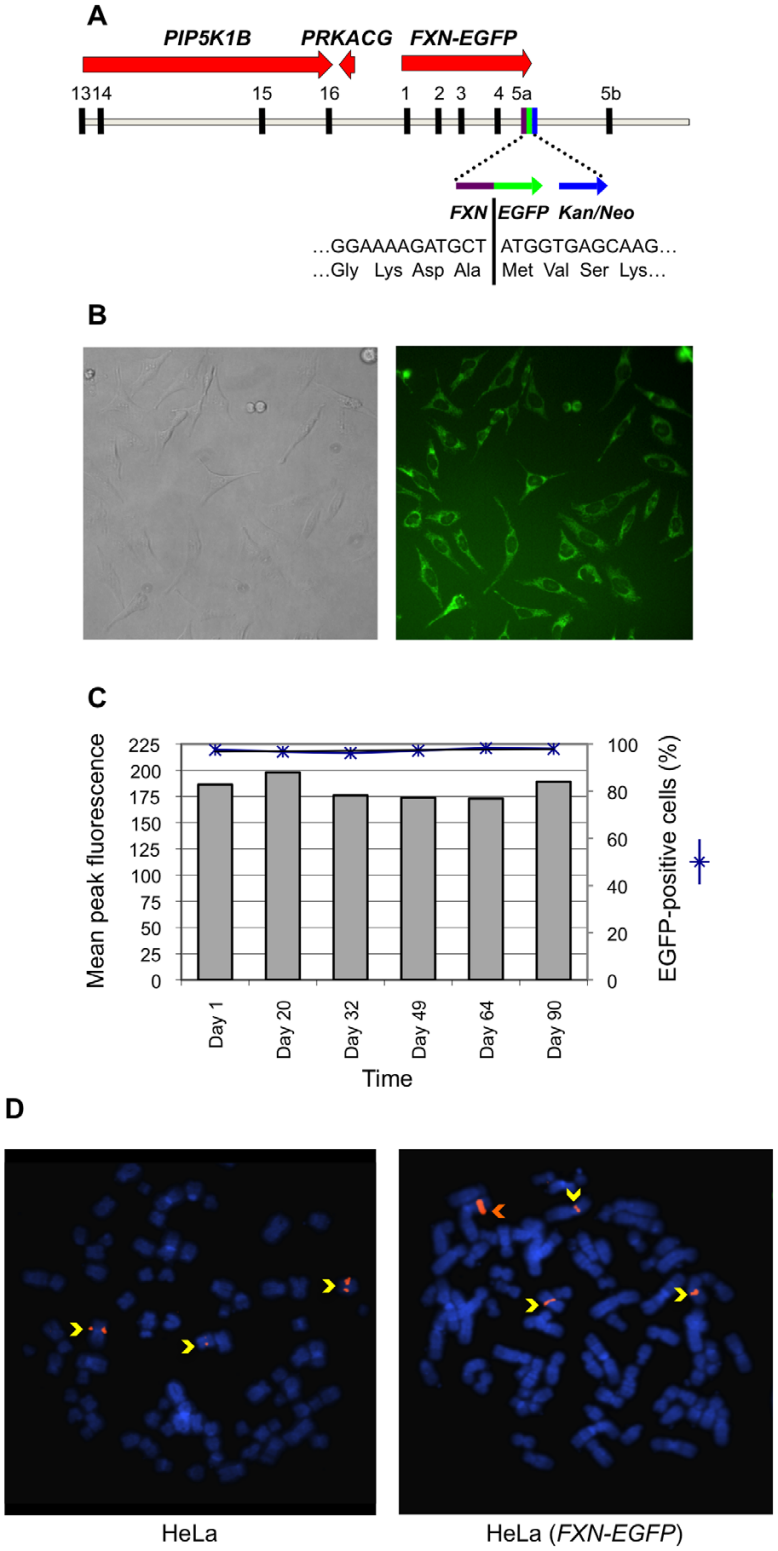
Characterization of HeLa (*FXN-EGFP*) stable cell lines. (A) Diagrammatic representation of the BAC genomic DNA fragment containing the *FXN-EGFP* genomic reporter construct. The sequence includes exons 13–16 of the *PIP5K1B* gene and the *PRKACG* gene upstream of the *FXN* locus, and about 23 kb of additional sequence downstream of exon 5b. The exon 5a–EGFP–Kan/Neo region is shown in greater detail. (B) Microscopic imaging. Transmitted light (left) and fluorescence (right) images of a HeLa (*FXN-EGFP*) stable cell line. EGFP expression produced by the *FXN-EGFP* genomic reporter is evident in all cells. (C) Flow cytometric analysis. The levels of EGFP expression (left Y-axis) and the proportion of EGFP-positive cells (right Y-axis) were stable following growth in continuous culture. (D) Determination of transgenic fragment integration site by FISH. Rhodamine-labeled RP11-265B8 was hybridized onto metaphase chromosomes (DAPI stained) of HeLa (left) and HeLa (*FXN-EGFP*) (right) cells. Three hybridization signals (yellow arrows) corresponded to the endogenous *FXN* gene. The presence of one additional brighter signal (orange arrow) establishes the presence of a single integration site containing multiple copies of the *FXN-EGFP* transgene.

We previously generated an *FXN-EGFP* genomic reporter fusion by using homologous recombination [43] to insert an EGFP-Kan/Neo cassette in-frame immediately following the final codon of exon 5a of the normal human *FXN* gene present on the 188 kb BAC clone RP11-265B8 [34]. The modified genomic insert from the BAC clone (**Figure 1A**) was isolated from most of the vector sequence by digestion at unique sites with *Asc*I and *Bsi*WI and transfected into HeLa cells. A number of stable cell lines were produced and selected for the Kan/Neo resistance determinant using G418, followed by limiting dilution to establish single cell clones (**Figure 1B**). Examination of a number of independent stable clones by flow cytometry indicated that the proportion of EGFP-positive cells in each clone was greater than 96%. These clones exhibited a tight fluorescence peak, indicating high homogeneity of the cell population, and varying values of median peak fluorescence (data not shown). The proportion of EGFP-positive cells and the relative levels of EGFP expression were found to be stable following growth in continuous culture for over 90 days (25 passages) (**Figure 1C**). Fluorescent *in situ* hybridization (FISH) using the entire RP11-265B8 sequence as a probe was performed on metaphase spreads of a selected HeLa (*FXN-EGFP*) stable cell line. HeLa cells have altered ploidy and three hybridization signals were observed corresponding to the endogenous *FXN* gene. The stable clone displayed an additional and brighter hybridization signal establishing the presence of a single integration site containing multiple copies of the *FXN-EGFP* transgene (**Figure 1D**).

### Primary Compound Screening Procedures

**Figure 2:**
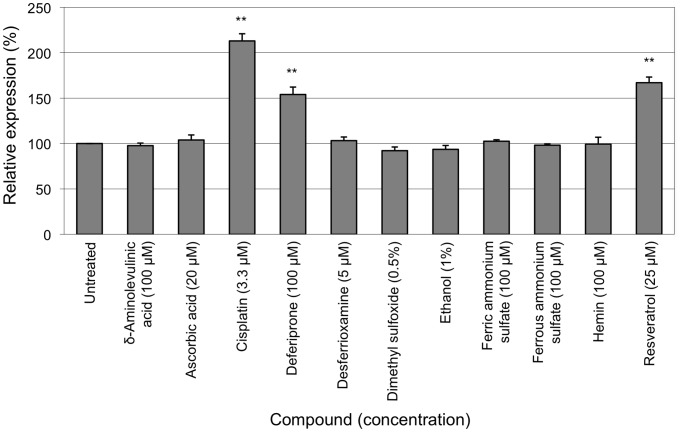
Primary compound screen. The HeLa (*FXN-EGFP*) stable cell line was exposed to various concentrations of selected test compounds for 72 hours. The levels of EGFP expression were measured by flow cytometry. For each compound the lowest concentration that produced the greatest change in *FXN-EGFP* expression is shown. Assays were performed in triplicate on at least three independent occasions. Error bars represent standard error of the mean. **p<0.01 in comparison to the untreated control.

A small-scale non-automated primary screen was conducted in which selected compounds were evaluated over a 10,000-fold concentration range on HeLa (*FXN-EGFP*) cells and EGFP fluorescence was measured by flow cytometry. Compounds were selected based on the known pathophysiology of FRDA including those involved in iron metabolism and antioxidants. Compounds were dissolved in water, ethanol or dimethyl sulfoxide (DMSO), as appropriate. None of the solvents had a significant effect on cell viability or the expression of the *FXN-EGFP* reporter at the concentrations used in the cellular assay (**Figure 2**). The anti-cancer compound cisplatin (3.3 µM) elicited a 2.2-fold increase in *FXN-EGFP* expression. The iron-chelator deferiprone (100 µM) was found to increase expression by 1.6-fold and the phytoalexin resveratrol (25 µM) increased expression by 1.7-fold. The combination of any two of these three compounds did not result in an additive effect on *FXN-EGFP* expression (data not shown). The other compounds tested had no notable effect on the level of *FXN-EGFP* expression.

### High Throughput Screening Procedures

The HeLa (*FXN-EGFP*) cellular genomic reporter assay was adapted for use in high throughput screening procedures. Cells were cultured in 96-well optical-quality microtiter plates and measurement of EGFP levels was via a fluorometer. To correct for well to well variations in cell number a measurement of cell viability was incorporated. HTS procedures were undertaken at the Walter and Eliza Hall Institute High-Throughput Chemical Screening Facility. The Spectrum Collection was screened which is a library containing 2,000 biologically active and structurally diverse compounds that are primarily FDA-approved compounds and some natural products. Each test compound was evaluated at a final concentration of 10 µM. Fluorometric measurement of EGFP fluorescence was performed using a bottom-plate reader followed by cell viability assays. A standard curve of cell number versus viability was also established for each run. The average background fluorescence of parental HeLa cells was subtracted from the EGFP readings of HeLa (*FXN-EGFP*) cells exposed to the chemical library. A correction factor for the number of viable cells was applied to the EGFP levels in each corresponding well. This was compared to the average value obtained from cells not exposed to any test compound to determine the level of change in *FXN* gene expression.

**Figure 3:**
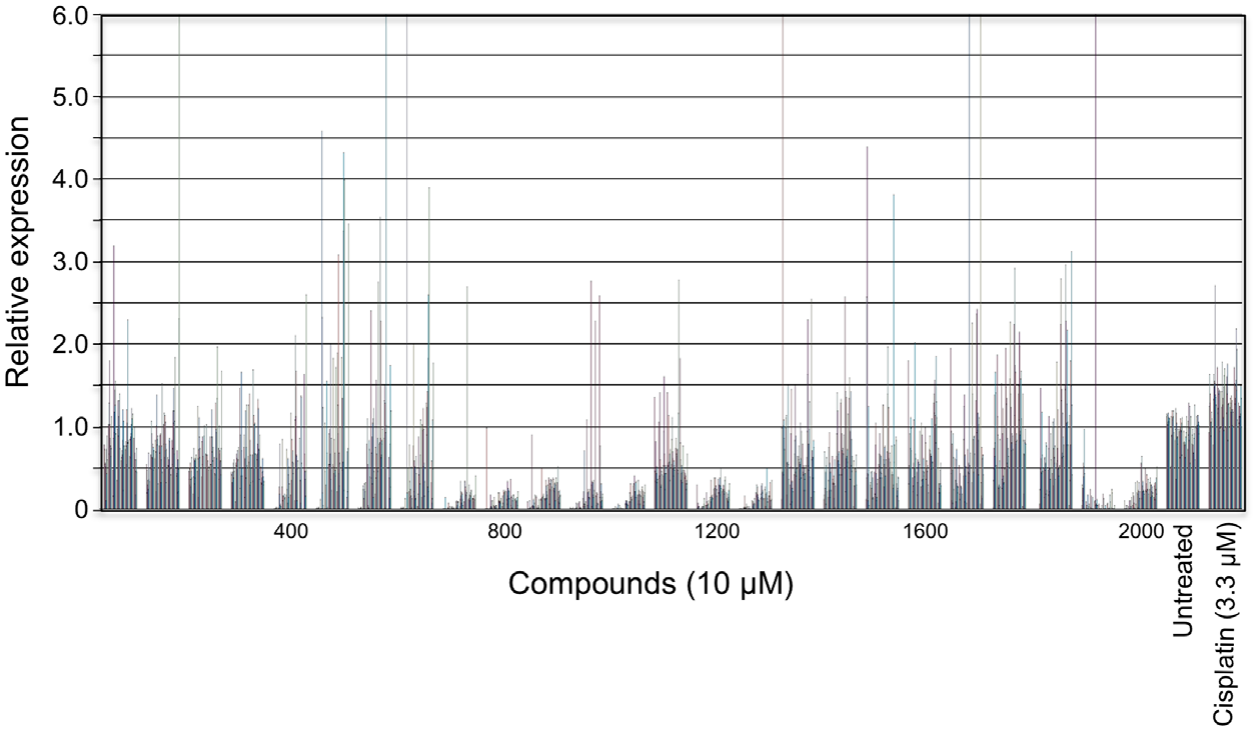
High throughput screening. The HeLa (*FXN-EGFP*) stable cell line was used in high throughput screening procedures to screen the Spectrum Collection compound library. Each test compound was evaluated at a final concentration of 10 µM. Controls included untreated cells and cells exposed to 3.3 µM cisplatin. Cultures were incubated for 72 hours. The fluorometric measurement of EGFP fluorescence was performed followed by the fluorometric measurement of cell viability. The average background fluorescence of parental HeLa cells was subtracted from the EGFP readings of HeLa (*FXN-EGFP*) cells exposed to the chemical library. A correction factor for the number of viable cells was applied to the EGFP levels in each corresponding well. This was compared to the average value obtained from cells not exposed to any test compound to determine the level of change in *FXN* gene expression.

Following the complete screening of the 2,000 compound library, 18 hit compounds were identified that elicited a greater than three-fold increase in *FXN-EGFP* expression at a concentration of 10 µM in the HeLa (*FXN-EGFP*) genomic reporter assay (**Figure 3**). A total of 74 compounds were identified that elicited a greater than 1.5-fold increase in EGFP expression (30 compounds increased expression by 1.5–2-fold; 15 by 2–2.5-fold; and 11 by 2.5–3-fold).

**Figure 4:**
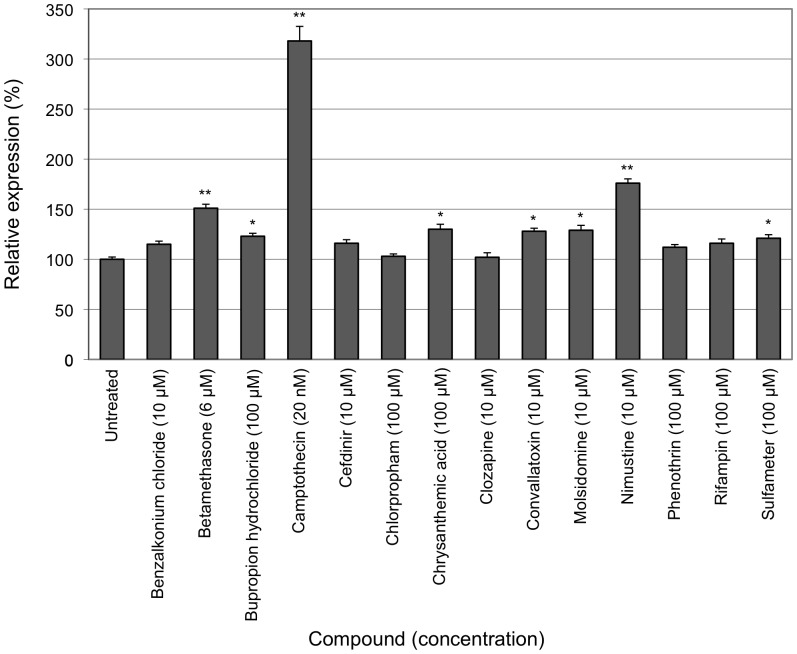
High throughput screening compound validation. The HeLa (*FXN-EGFP*) stable cell line was exposed to various concentrations of compounds identified by high throughput screening. Cultures were incubated for 72 hours. The levels of EGFP expression were measured by flow cytometry. For each compound the lowest concentration that produced the greatest change in *FXN-EGFP* expression is shown. Assays were performed in triplicate on at least three independent occasions. Error bars represent standard error of the mean. *p<0.05, **p<0.01 in comparison to the untreated control.

Most of the primary hit compounds that increased expression by greater than 1.5-fold that were available for commercial purchase or closely related analogs that were available were subjected to dose-response experiments over a large concentration range on HeLa (*FXN-EGFP*) cells and EGFP fluorescence was measured by flow cytometry. Three compounds were validated to reproducibly increase EGFP levels by greater than 1.5-fold in the flow cytometric analysis (**Figure 4**). The greatest level of induction was observed with the anti-cancer compound camptothecin, which elicited a 3.3-fold increase in *FXN-EGFP* expression at a concentration of 20 nM. This is the greatest level of induction that we have observed with the HeLa (*FXN-EGFP*) genomic reporter assay. The glucocorticoid betamethasone (6 µM) and the alkylating agent nimustine (10 µM) increased *FXN-EGFP* expression by 1.5-fold and 1.7-fold, respectively. Bupropion hydrochloride (100 µM), chrysanthemic acid (100 µM), convallatoxin (10 µM), molsidomine (10 µM) and sulfameter (100 µM) elicited modest (less than 1.5-fold) but significant levels of induction of *FXN-EGFP* expression.

### Compound Evaluation in Friedreich Ataxia Cells

The *FXN-EGFP* genomic reporter contains a normal copy of the *FXN* gene without a GAA trinucleotide repeat expansion. Selected compounds were therefore subsequently evaluated on cells derived from individuals with FRDA. Transformed lymphoblasts and primary fibroblasts containing a GAA trinucleotide expansion on both *FXN* alleles were used. The levels of *FXN* mRNA were measured by quantitative real time RT-PCR and the levels of frataxin protein were measured by lateral flow immunoassay (dipstick assay).

**Figure 5:**
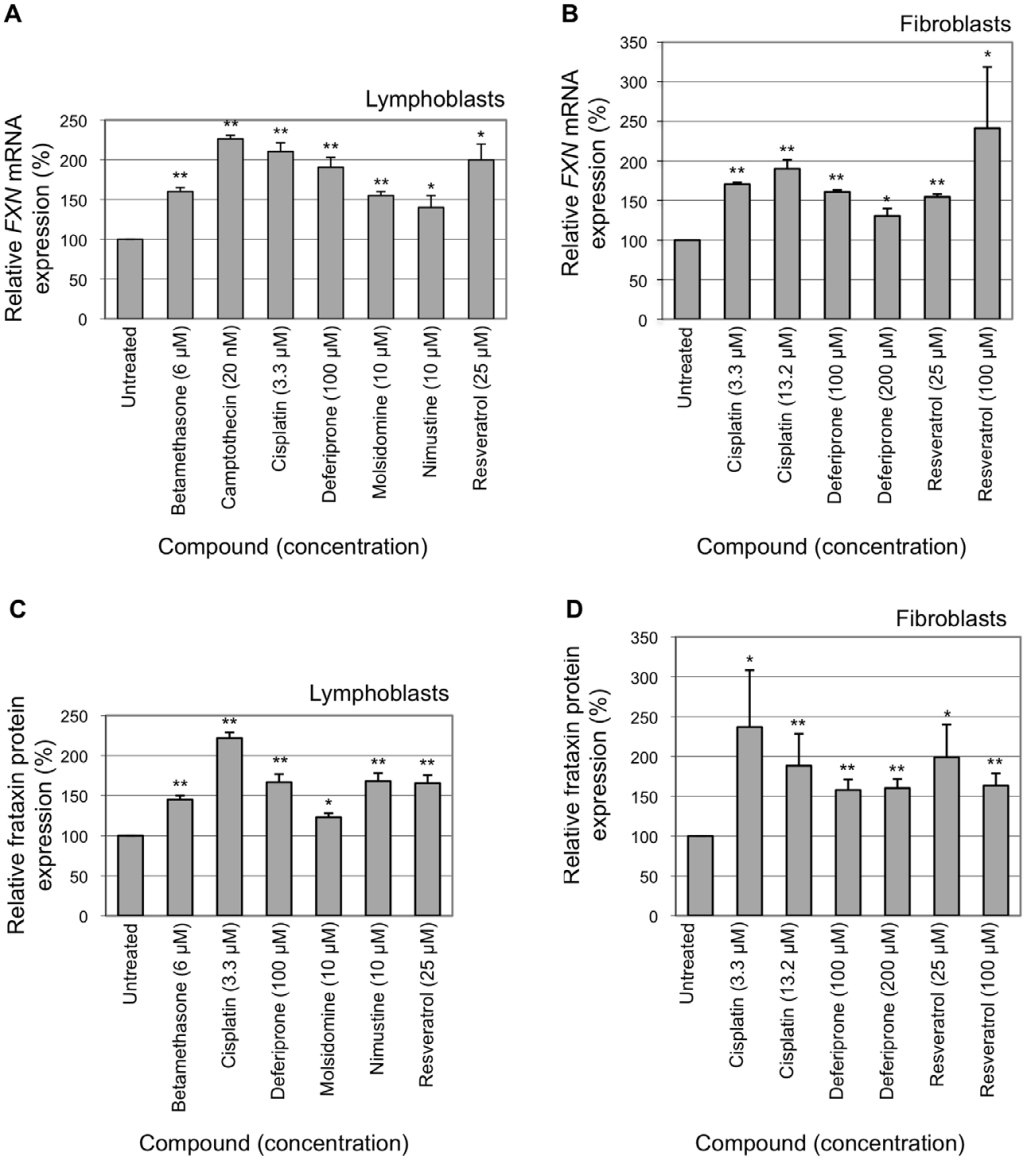
Compound evaluation in Friedreich ataxia cells. The modulation of *FXN* gene expression by selected test compounds was evaluated in lymphoblast and fibroblast cell lines derived from individuals with FRDA. All cell lines were homozygous for a GAA repeat expansion in the first intron of the *FXN* gene. Cultures were incubated for 72 hours. *FXN* mRNA levels were measured by real-time RT-PCR. Frataxin protein levels were measured by lateral flow immunoassay. (A) *FXN* mRNA levels measured in FRDA lymphoblast cell lines. (B) *FXN* mRNA levels measured in FRDA fibroblast cell lines. (C) Frataxin protein levels measured in FRDA lymphoblast cell lines. (D) Frataxin protein levels measured in FRDA fibroblast cell lines. Assays were performed in triplicate on at least three independent occasions. Error bars represent standard error of the mean. *p<0.05, **p<0.01 in comparison to the untreated control.

The anti-cancer compounds cisplatin (3.3 µM) and camptothecin (20 nM) were found to increase the level of *FXN* mRNA by over 2-fold in FRDA lymphoblasts. Resveratrol (25 µM) increased mRNA levels by 2-fold in FRDA lymphoblasts. Betamethasone (6 µM), deferiprone (100 µM) and molsidomine (10 µM) elicited increases in the level of *FXN* mRNA by over 1.5-fold in FRDA lymphoblasts (**Figure 5A**). Cisplatin (13.2 µM) and deferiprone (100 µM) were found to increase the level of *FXN* mRNA by over 1.5-fold in FRDA fibroblasts. Resveratrol (100 µM) elicited a 2.4-fold increase in *FXN* mRNA levels in FRDA fibroblasts (**Figure 5B**).

Cisplatin (3.3 µM) was found to increase the levels of frataxin protein by 2.2-fold in FRDA lymphoblasts. Deferiprone (100 µM), nimustine (10 µM) and resveratrol (25 µM) each increased the levels of frataxin protein by1.7-fold in FRDA lymphoblasts. Betamethasone (6 µM) elicited a 1.4-fold increase in frataxin levels in FRDA lymphoblasts (**Figure 5C**). Cisplatin (3.3 µM), deferiprone (100 µM) and resveratrol (25 µM) increased frataxin protein levels by 2.4-fold, 1.6-fold and 2-fold, respectively, in FRDA fibroblasts (**Figure 5D**).

### Compound Evaluation in a Friedreich Ataxia Mouse Model

As resveratrol is a safe drug and has a number of proposed modes of action that may be beneficial in FRDA including neuroprotection, cardioprotection and antioxidant properties, an *in vivo* evaluation of this compound was conducted in a YAC-based GAA expansion humanized mouse model of FRDA [44]. The YG8R transgenic mice contain the entire human genomic *FXN* gene containing a GAA repeat expansion in the first intron of the gene, and also contain a homozygous knockout of the murine *Fxn* gene. Only transgenic human frataxin protein is expressed in the mice. The mice provide the same underlying molecular cause of the disease as found in individuals with FRDA and exhibit decreased frataxin levels, progressive neuronal and cardiac pathology that recapitulates that seen in individuals with FRDA and measurable neurobehavioral deficits consistent with that observed in FRDA [41].

**Figure 6:**
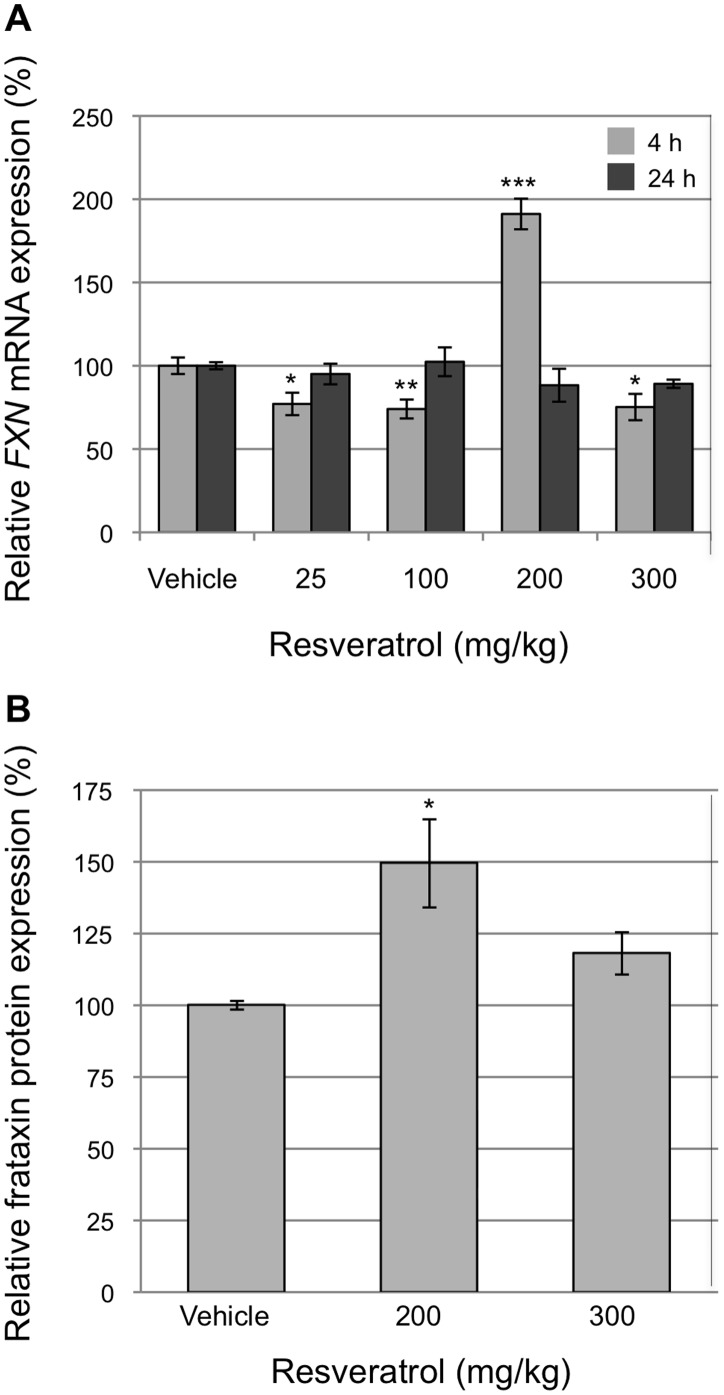
Evaluation of resveratrol in a Friedreich ataxia mouse model. The modulation of *FXN* gene expression by resveratrol was evaluated in the YG8R humanized FRDA mouse model. Resveratrol was administered daily for three days by subcutaneous injection. (A) The levels of human *FXN* mRNA were measured by real-time RT-PCR. Brain samples were collected at four and 24 hours after the final dose. (B) The levels of human frataxin protein were measured by lateral flow immunoassay. Brain samples were collected four hours after the final dose. Reactions were carried out in triplicate for each biological sample (n = 4). Error bars represent standard error of the mean. *p<0.05, **p<0.01, ***p<0.001 in comparison to the untreated control (vehicle).

Resveratrol was administered daily for three days by subcutaneous injection at doses ranging from 25–300 mg/kg. Samples were collected at four and 24 hours after the final dose. The levels of human *FXN* mRNA were measured by real-time RT-PCR. An increase in human *FXN* gene expression of almost 2-fold was observed in mouse brain at a dose of 200 mg/kg (**Figure 6A**). The levels of human frataxin protein were measured by lateral flow immunoassay. Human frataxin protein was found to increase by 1.5-fold in transgenic mouse brain at the same dose (**Figure 6B**), indicating the ability of resveratrol to cross the blood-brain barrier and to exert a frataxin-inducing effect. Interestingly, no other dose of resveratrol resulted in an increase in *FXN* mRNA or frataxin protein levels in mice and some doses resulted in a reduction in mRNA.

## Discussion

The clinical manifestations of FRDA are due to the presence of a GAA trinucleotide repeat expansion within the first intron of the *FXN* gene that results in decreased gene expression and an insufficiency of frataxin protein. An overall inverse correlation exists between the length of the smaller GAA repeat expansion and the level of *FXN* transcript, the amount of frataxin protein and the age of onset of disease symptoms [29]–[33]. Individuals who are heterozygous for a GAA repeat expansion produce about half the normal level of frataxin and are asymptomatic. The GAA repeat expansion mutation does not alter the coding sequence of the gene and the low levels of frataxin protein produced retain normal function. In theory, any increase in frataxin levels should prove beneficial, while a several-fold increase could be sufficient to halt disease progression. Initiation of treatment at the onset of symptoms or prior to onset has the potential to delay or prevent the manifestation of disease pathology. The pharmacological up-regulation of *FXN* gene expression is a therapeutic approach for the treatment of FRDA that directly addresses the primary issue of frataxin deficiency rather than secondary disease effects.

In order to rapidly and accurately monitor human *FXN* gene expression and evaluate compounds for their ability to modulate the expression of the gene, we developed an *FXN-EGFP* genomic reporter that consists of the fusion of the EGFP gene in-frame immediately following the final codon of exon 5a of the normal human *FXN* gene present on a BAC clone [34]. Genomic reporters preserve the normal location and spacing of many regulatory elements that may be positioned over large distances in the surrounding chromosomal region and more accurately recapitulate expression patterns of tagged genes than can be obtained with conventional small vector transgenic constructs. We have now developed a cellular genomic reporter assay for *FXN* gene expression by the generation of a stable HeLa cell line containing the *FXN-EGFP* genomic reporter that permits the precise measurement of human *FXN* gene expression by flow cytometry and fluorometry.

The *FXN-EGFP* reporter construct could be stably maintained and expressed in HeLa cells. We did not observe reduced cell viability, reduction in fluorescence or variegation of expression in the stable cell line after repeated passages of cell cultures. The HeLa cell line was selected because a robust and rapidly growing human cell type with undemanding growth conditions was required for HTS procedures. As the *FXN* gene appears to be expressed in all cell types analyzed we did not believe that it was necessary to introduce the *FXN-EGFP* genomic reporter into cell lines derived from tissues affected in the disorder, namely neuronal and cardiac cells. In addition, the more fastidious growth requirements of such cell types would negatively impact on the feasibility of HTS procedures.

EGFP expression in the cellular assay is under the control of the natural *FXN* promoter and regulatory elements present on the BAC clone. As the *FXN* gene is not highly expressed the levels of EGFP expressed by the *FXN-EGFP* genomic reporter were relatively low. EGFP expression was sufficiently robust for accurate and reproducible measurement by flow cytometry but required careful optimization of the fluorometric detection system used in HTS procedures. The specific HeLa (*FXN-EGFP*) stable cell line that was selected for compound screening procedures produced the highest levels of mean peak fluorescence among the clones isolated and was shown by FISH analysis to contain multiple copies of the *FXN-EGFP* transgene at a single integration site. This clone was used in order to maximize the signal-to-noise ratio.

An advantage of using EGFP as a readout of gene expression is that EGFP reporter expression can be visualized in living cells without the need for cell fixation, cell disruption or addition of enzymatic substrates. For flow cytometry purposes, cells are collected and readings are made on a per cell basis. For HTS procedures the detection of EGFP fluorescence can be made *in situ*, but HeLa cells and derivatives grow as a monolayer attached to the bottom of culture vessels and the EGFP signal is not uniformly distributed in solution throughout each well. It was therefore necessary to adapt the optics of the plate reader to make measurements from beneath the growth chamber focused on a plane corresponding to the cell monolayer. Optical-grade microtiter plates were necessary in order to minimize quenching of the signal read through the base of the plates. In order to account for the possible non-uniform growth of cells exposed to certain compounds, readings were taken at multiple positions for each well. To increase the signal-to-noise ratio in future screens the assay system could be modified to express an alternative reporter with greater signal intensity or by using a two-step transcriptional amplification (TSTA) system [45] to amplify the signal.

Changes in frataxin-EGFP content per cell in response to exposure to pharmacological compounds may reflect not only changes at the transcriptional level, but also changes in the processing and export of mRNA and in mRNA and protein turnover. This is a significant advantage of the *FXN-EGFP* assay system, since changes in any one of these processes could affect frataxin levels in a therapeutic manner. It is possible that the induction of frataxin expression quantified by the *FXN-EGFP* cellular genomic reporter assay may be underestimated due to turnover of the fusion protein during the induction period. EGFP has been reported to have a half-life of about 26 hours [46] but the half-life of the frataxin-EGFP fusion protein has not been determined. Cells are typically incubated with test compounds for a period of 72 hours in the assay system. The effects of compounds with a short half-life may be diminished at the time of readout. Some of these issues could be alleviated by replenishing cultures with fresh test compound periodically throughout the assay incubation period or taking more frequent readings. However, these modifications would increase the complexity and costs of HTS procedures.

The level of increase in EGFP expression induced by the positive control drug cisplatin quantified via fluorometric measurement in the HTS assay (1.4-fold) was lower than that measured via flow cytometry (2.2-fold). This likely represents inherent differences in the quantification of fluorescence by the two detection techniques and could contribute to false negative readouts. Other factors leading to potential false negative results include compounds causing complete cell death at the tested concentration (10 µM) or those not sufficiently potent at this concentration. False positive readouts could be attributed to compounds that were intrinsically fluorescent or to stochastic variability inherent in the HTS assay that was performed a single time. Post-HTS verification evaluation identified false positive compounds and excluded them from further consideration.

The entire 2,000 compound Spectrum Collection library was subjected to HTS procedures. The relatively small size of the chemical library made it cost-effective and suitable for use as an initial proof-of-principle screen. In addition, as the majority of the compounds in this library are FDA-approved, it was envisaged that promising hit compounds could be more rapidly moved into a clinical setting. The screening of such a library also lends itself to the Selective Optimization of Side Activities (SOSA) approach in which known pharmacological agents are structurally modified to give increased affinity for a new target [47].

Of therapeutic relevance, it is important to underscore that although the *FXN-EGFP* genomic reporter contains a normal copy of the *FXN* gene without a GAA trinucleotide repeat expansion, its use has allowed the identification of a number of agents that are capable of increasing *FXN* gene expression and frataxin protein in FRDA cells. However, classes of compounds that are specifically able to overcome the effects of the GAA repeat expansion on *FXN* gene expression would not be detected using this assay. This limitation could be overcome by the development of an *FXN* genomic reporter construct containing a long GAA repeat expansion.

Two anticancer agents, cisplatin and camptothecin, were found to elicit the highest levels of induction in the HeLa (*FXN-EGFP*) genomic reporter and in FRDA lymphoblasts and fibroblasts. Cisplatin is a platinum-containing compound that binds to and cross-links DNA most commonly acting at adjacent guanine residues [48]. It was previously shown to increase *FXN* gene expression in a cisplatin-resistant ovarian carcinoma cell line [49]. Camptothecin is a cytotoxic quinoline alkaloid that inhibits DNA topoisomerase I [50]. The mechanisms of action that modulate *FXN* gene expression are not known but likely due to non-specific effects. Both drugs have toxic side-effects that limit their use for the long-term treatment of FRDA.

Deferiprone is a membrane-permeable iron-chelator that has the ability to shuttle iron between cellular organelles and across membranes [51]. Deferiprone binds to ferric ions to form neutral 3∶1 (deferiprone:iron) complexes. It is in clinical use for the treatment of thalassemia. In an open-label clinical trial examining individuals with FRDA deferiprone appeared to reduce accumulated iron in the dentate nucleus and reduced neuropathy and ataxic gait [35]. Our findings indicate that deferiprone is also able to induce *FXN* gene expression and increase the levels of frataxin suggesting that intracellular iron levels can modulate *FXN* gene regulation. Besides the direct effects of deferiprone on iron mobilization in FRDA it may confer the additional benefit of increased frataxin levels.

A number of compounds were found to elicit modest but measurably reproducible increases in *FXN-EGFP* expression. These include: bupropion, a norepinephrine-dopamine reuptake inhibitor used as an antidepressant; chrysanthemic acid, a compound related to pyrethroids; onvallatoxin, a glycoside used in the treatment of cardiac conditions; and sulfameter, a long-acting sulfonamide antibacterial. Additional compounds that were identified to result in small increases in *FXN* gene expression and frataxin protein in FRDA cells include: betamethasone, a glucocorticoid with anti-inflammatory and immunosuppressive properties; nimustine, a nitrosourea alkylating agent with antineoplastic activity; and molsidomine, an orally active and long acting vasodilating drug.

Resveratrol was identified in the HeLa (*FXN-EGFP*) cellular genomic reporter to increase *FXN* gene expression by 1.7-fold. It was subsequently shown to increase *FXN* mRNA levels by 2-fold in FRDA lymphoblasts and by 2.4-fold in FRDA fibroblasts. It elicited an increase in frataxin protein by 1.7-fold in FRDA lymphoblasts and by 2-fold in FRDA fibroblasts. Resveratrol (3,4′,5-trihydroxy-trans-stilbene) is a polyphenolic compound that is synthesized naturally by several plant species. It is commonly found in red grape skin, berries and nuts [52]. Resveratrol is a phytoalexin; a toxic compound produced by higher plants in response to infection or other stresses. Resveratrol appears to have a wide spectrum of targets as a pharmacological agent. It has been proposed as a treatment for cancers, cardiac disease, neurodegenerative conditions and metabolic disorders including diabetes [53], [54]. It has also been reported to possess antioxidant, anti-inflammatory, anti-angiogenic and antiviral properties. There is evidence that resveratrol contributes to enhanced longevity in *S. cerevisiae* [55], *C. elegans* [56], *D. melanogaster* [56] and mice consuming a high caloric diet [57]. The effects on life extension appear to mimic several of the biochemical effects of calorie restriction.

There is divergent evidence regarding the ability of resveratrol to interact with sirtuin proteins. Sirtuins are NAD^+^-dependent deacetylases that have been implicated to influence aging, transcription regulation, apoptosis and stress resistance. The deacetylase activity of sirtuins is directed to histone proteins and transcription factors including p53, FOXO proteins and the peroxisome proliferator activated receptor gamma (PPAR-γ) coactivator 1 alpha (PGC-1α) [58]. There are seven sirtuin genes in humans (*SIRT1-7*). There is some debate regarding the ability of resveratrol to directly activate Sirtuin 1 or increase expression of the *SIRT1* gene [53], [55], [59], [60].

PGC-1α plays a role in the regulation of mitochondrial function and fatty acid oxidation [61]. It is activated by deacetylation by Sirtuin 1 which has also been shown to repress PPAR-γ [62]. There is evidence of cross regulation mechanisms that act between frataxin and PGC-1α. Reduced expression of PGC-1α has been shown in FRDA cells, in several tissues of an FRDA mouse model and upon inhibition of *FXN* gene expression by shRNA [63]. The downregulation of PGC-1α by siRNA results in decreased *FXN* transcript and frataxin protein in human control and FRDA fibroblasts [64]. Conversely, the overexpression of PGC-1α in skeletal muscle cells has been shown to increase frataxin levels [65], and the PPAR-γ agonist Azelaoyl PAF is able to increase *FXN* gene expression and frataxin protein levels in FRDA cells [66]. These findings suggest a mechanism that accounts for the observed increased levels of *FXN* gene expression and frataxin protein levels mediated by resveratrol. It can be hypothesized that the activation of Sirtuin 1 by resveratrol enables it to modulate the activity of the PPAR-γ and PGC-1α pathway which in turn influences the regulation of *FXN* gene expression.

Resveratrol has a good safety profile and has not been reported to have any major adverse side-effects in humans [67]. Of the pharmacological compounds that we identified to increase *FXN* gene expression, resveratrol had the most favorable safety criteria for human use and is expected to be well tolerated by individuals with FRDA. It is also able to cross the blood-brain barrier [68], [69]. Pharmacokinetic studies indicate that the metabolization of resveratrol into conjugated forms may influence its bioavailability in higher organisms [70], [71]. We therefore further evaluated resveratrol pre-clinically in the YG8R humanized mouse model of FRDA that contains the entire human *FXN* gene incorporating a GAA trinucleotide repeat expansion. At a dose of 200 mg/kg resveratrol was shown to increase human *FXN* gene transcription by almost 2-fold and frataxin protein was found to increase by 1.5-fold in transgenic mouse brain. It is curious that this was the only dose at which *FXN* mRNA and frataxin protein were increased. On the basis of this data, an open-label, proof of principle study of resveratrol in individuals with FRDA is currently underway.

In conclusion, we have demonstrated the utility of using an *FXN-EGFP* cellular genomic reporter assay in HTS procedures for the identification of pharmacological compounds able to increase *FXN* gene expression and frataxin protein. Any compound that specifically increases frataxin levels by several-fold in individuals with FRDA could serve as a potential pharmacological therapy for the disorder. Candidate agents were processed along the drug development pipeline from drug screening to lead identification and resveratrol was selected for evaluation in pre-clinical animal testing and is currently being evaluated in a human therapeutic trial. Ultimately, a cocktail of drugs may be required to provide an effective therapy for FRDA. This may include pharmacological agents that address the primary defect of frataxin deficiency by increasing *FXN* gene expression and/or frataxin protein such as those identified in this study, HDAC inhibitors [72]–[74], erythropoietin [75]–[77], PPAR-γ agonists [66] or IFNγ [78], and those directed at secondary effects, such as antioxidants and iron-chelators.
